# Inhibition of Tyrosinase and Melanogenesis by a White Mulberry Fruit Extract

**DOI:** 10.3390/ijms26157589

**Published:** 2025-08-06

**Authors:** Nuttawadee Prasawang, Nareerat Sutjarit, Athisri Sitthipunya, Prasit Suwannalert, Wutarak Monsuwan, Nisamanee Charoenchon

**Affiliations:** 1 Department of Pathobiology, Faculty of Science, Mahidol University, Bangkok 10400, Thailand; nattawadee.psw@gmail.com (N.P.); athisri.sit@mahidol.ac.th (A.S.); prasit.suw@mahidol.ac.th (P.S.); 2 Nutrition Unit, Faculty of Medicine Ramathibodi Hospital, Mahidol University, Bangkok 10400, Thailand; nareerat.sut@mahidol.ac.th (N.S.); wutarak.pue@mahidol.edu (W.M.)

**Keywords:** mulberry crude extract, UVB radiation, melanogenesis, B16F10 cells, zebrafish embryo, tyrosinase inhibition

## Abstract

Ultraviolet B (UVB) radiation is a key factor in the overproduction of melanin in the skin. Melanocytes produce melanin through melanogenesis to protect the skin from UVB radiation-induced damage. However, excessive melanogenesis can lead to hyperpigmentation and increase the risk of malignant melanoma. Tyrosinase is the rate-limiting enzyme in melanogenesis; it catalyzes the oxidation of tyrosine to 3,4-dihydroxy-L-phenylalanine and subsequently to dopaquinone. Thus, inhibiting tyrosinase is a promising strategy for preventing melanogenesis and skin hyperpigmentation. White mulberry (*Morus alba* L.) is rich in antioxidants, and mulberry fruit extracts have been used as cosmetic skin-lightening agents. However, data on the capacity of mulberry fruit extracts to inhibit tyrosinase under UVB radiation-induced melanogenic conditions remain scarce, especially in an in vivo model. In this study, we evaluated the effects of a mulberry crude extract (MCE) on UVB radiation-induced melanogenesis in B16F10 melanoma cells and zebrafish embryos. The MCE significantly reduced tyrosinase activity and melanogenesis in a dose-dependent manner without inducing cytotoxicity. These effects are likely attributable to the antioxidant constituents of the extract. Our findings highlight the potential of this MCE as an effective tyrosinase inhibitor for the prevention of UVB radiation-induced skin hyperpigmentation.

## 1. Introduction

Hyperpigmentation is a common dermatological condition characterized by the darkening of the skin due to excessive melanin production or deposition [1]. Although not life-threatening, it can significantly impact quality of life by causing emotional distress, lowered self-esteem, and social stigma [2]. The underlying cause of hyperpigmentation involves a process called melanogenesis, in which melanocytes, located in the basal layer of the epidermis, produce melanin. A key enzyme in this process is tyrosinase, which catalyzes crucial steps in melanin production [3]. Melanogenesis can be influenced by both intrinsic and extrinsic factors. Intrinsic factors include genetic predisposition and hormonal fluctuations, while extrinsic triggers—such as UV radiation, inflammation, and certain medications—can aggravate pigmentation irregularities [4,5]. Notably, epidemiological data indicate that prolonged UV exposure is a major contributor to hyperpigmentation, skin damage, and skin cancer, particularly in tropical regions with high UV indices [6,7]. Global warming and ozone depletion further exacerbate this issue by increasing UV exposure worldwide [8].

Among UV radiation types, UVB is the most cytotoxic and mutagenic, causing direct DNA damage in keratinocytes and stimulating melanogenesis [9]. The negative effects of UVB radiation on the skin are primarily due to its capacity to increase melanin production, which occurs via the process of melanogenesis. UVB exposure promotes the release of α-melanocyte-stimulating hormone (α-MSH), which activates the transcription factor MITF (microphthalmia-associated transcription factor), a key regulator of melanocyte survival and melanin biosynthesis [10]. MITF upregulates melanogenic genes, including tyrosinase, TRP-1, and TRP-2. Tyrosinase, the rate-limiting enzyme, hydroxylates monophenols to o-diphenols and oxidizes them to o-quinones, which subsequently polymerize into melanin [11]. In addition to the α-MSH/MC1R/MITF axis, studies suggest that UVB can directly stimulate melanocytes, such as B16F10 cells, through oxidative stress-mediated pathways, leading to increased melanin synthesis [12]. Although increased melanin synthesis serves as a protective response against UV-induced DNA damage [13], excessive melanin production can lead to hyperpigmentation, which is a common dermatological concern.

Current treatments for hyperpigmentation typically involve tyrosinase inhibitors (e.g., hydroquinone, kojic acid), antioxidants, and UV-protective agents. However, many synthetic compounds are associated with adverse effects such as irritation, erythema, and paradoxical hyperpigmentation, including blue melasma [14,15]. This underscores the need for safer, natural alternatives with both antioxidant and anti-melanogenic properties. White mulberry (*Morus alba* L.) is a deciduous plant widely distributed in temperate and subtropical regions. Extracts from its leaves, bark, roots, and fruits have demonstrated diverse biological activities and are extensively used in food, cosmetic, and nutraceutical products [16]. Notably, extracts from the leaves, bark, and roots possess strong anti-tyrosinase activity, primarily due to bioactive stilbenoids such as oxyresveratrol and mulberroside A [17,18,19]. These compounds chelate copper ions at the enzyme’s active site, thereby inhibiting the catalytic conversion of L-tyrosine to L-DOPA and dopaquinone—critical steps in melanin synthesis [16,20,21,22,23,24]. Accordingly, mulberry-derived extracts are often incorporated into skin-whitening products targeting pigmentation disorders [18]. While numerous studies have explored the bioactivity of white mulberry leaves and bark, relatively limited data exist on the effects of ripe *Morus alba* fruit extract on melanogenesis and tyrosinase activity.

Mulberry fruit extracts have been shown to have antioxidant activity and are mainly used in supplements and beverages [25,26]. Mulberry fruit extracts, particularly white mulberry, are predominantly valued for their antioxidant capacity, mainly attributed to high levels of anthocyanins, flavonols (e.g., quercetin, rutin), and phenolic acids (e.g., gallic acid, chlorogenic acid) [16]. These compounds exert free radical-scavenging activity by donating hydrogen atoms or electrons to neutralize reactive oxygen species (ROS), thereby mitigating oxidative stress implicated in skin aging and inflammation [27]. Moreover, anthocyanins such as cyanidin-3-glucoside, which is abundant in mulberry fruit, have demonstrated the ability to attenuate UVB-induced melanogenesis by downregulating MITF, tyrosinase, and related melanogenic genes in keratinocytes and melanocytes [28]. However, mulberry fruit extract is generally considered less effective as a skin-lightening agent compared to extracts derived from other parts of the plant, such as the root bark or leaves. This may be attributed, in part, to the limited number of studies investigating the anti-melanogenic effects of mulberry fruit extracts, particularly in an in vivo model.

This study addresses this gap by investigating the effects of ripe fruit extract in both B16F10 melanoma cells and zebrafish embryos. Our results provide supporting evidence for the anti-tyrosinase and anti-melanogenic properties of MCE, particularly in vivo model. These findings highlight the potential of MCE as a candidate for the development of skin-whitening agents. While preliminary, this study establishes a scientific foundation for future investigations, including clinical studies, to further explore the efficacy and safety of MCE in cosmetic or therapeutic applications.

## 2. Results

### 2.1. Free Radical Scavenging Activity and Phenolic Content of the MCE

The antioxidant activity and phenolic content of both the unfiltered and filtered MCE were evaluated to ensure that the standard filtration step commonly applied to sterilize samples before biological testing does not inadvertently remove or reduce key antioxidant or phenolic compounds. Paper or membrane filters may absorb low-molecular-weight polyphenols or precipitate suspended antioxidant particles. We used 2,2′-azinobis-3-ethylbenzothiazoline-6-sulfonic acid (ABTS), 2,2-diphenyl-1-picrylhydrazyl (DPPH), and ferric reducing antioxidant power (FRAP) assays to assess the free radical scavenging activity, and we used the Folin–Ciocalteu method to measure the total phenolic content. As shown in Table 1, there was no significant difference in the free radical scavenging activity and total phenolic content of the MCE before and after it was passed through the filter paper.

### 2.2. Phytochemical Composition of MCE 

**Figure 1:**
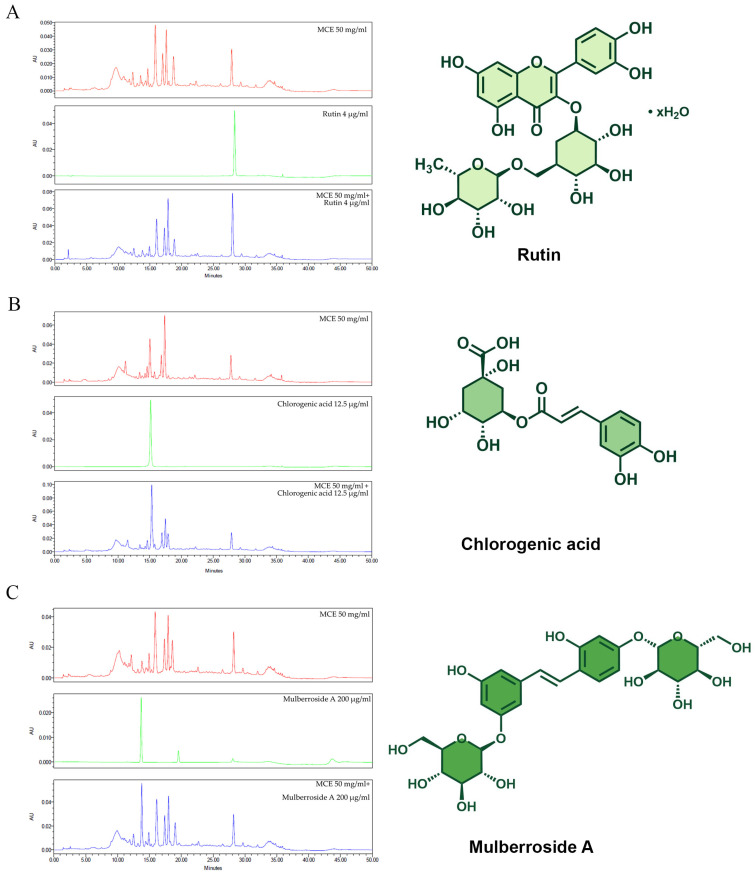
Comparison of MCE chromatogram with those of (**A**) rutin, (**B**) chlorogenic acid, and (**C**) mulberroside A, along with chemical structures of standard compounds. Chromatogram of MCE at 50 mg/mL is shown in red, chromatograms of standard compounds are shown in green, and chromatograms of MCE combined with each standard compound are shown in blue.

When we characterized the phytochemical composition of the MCE using high-performance liquid chromatography (HPLC), we found the anti-melanogenic components that were rutin, chlorogenic acid, and mulberroside A (Figure 1A–C). As shown in Table 2, the identity of these components was verified via HPLC analysis of 3 reference compounds: rutin (retention time: 28.35 min), chlorogenic acid (15.147 min), and mulberroside A (13.711 min). The presence of significant peaks corresponding to rutin, chlorogenic acid, and mulberroside A in MCE samples spiked with these compounds validated the identification of these phytochemicals within the MCE. Hence, three compounds with known anti-tyrosinase activity were detected in the MCE. MCE 50 mg/mL comprises 0.42 μg rutin, 491.77 μg chlorogenic acid, and 80.80 μg mulberroside A, as the main anti-melanogenic active compound.

### 2.3. Effect of the MCE on Cell Viability and the Effects of UVB Radiation on Cell Viability, Tyrosinase Activity, and Melanin Content

**Figure 2:**
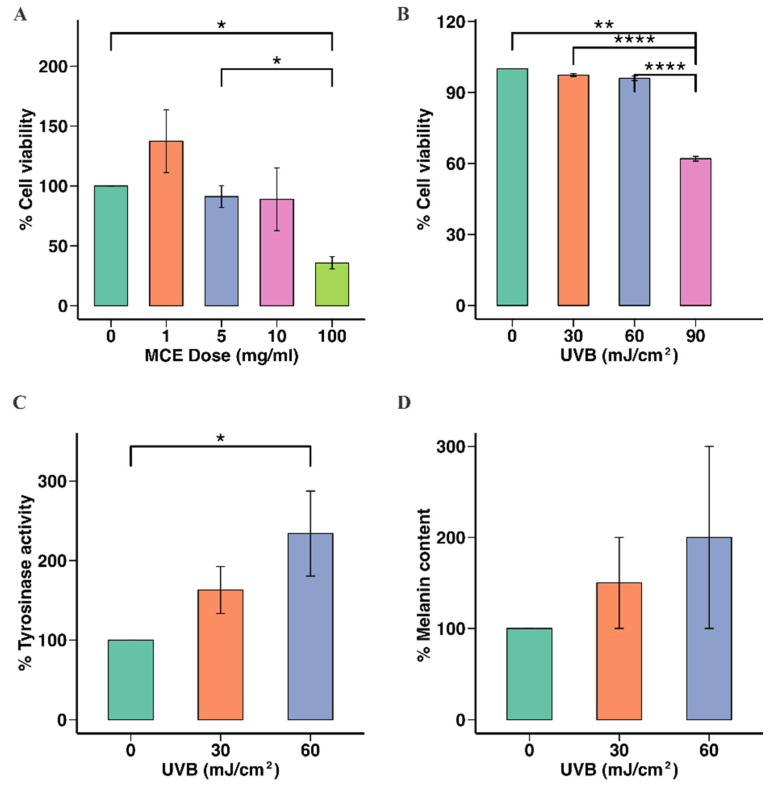
Effects of MCE and UVB radiation on B16F10 cell viability, tyrosinase activity, and melanin content. (**A**) Cytotoxic effect of MCE on B16F10 cells. (**B**) Effect of UVB radiation on B16F10 cell viability. (**C**) Effect of UVB radiation on cellular tyrosinase activity in B16F10 cells. (**D**) Impact of UVB radiation on melanin levels in B16F10 cells is presented as mean ± standard deviation. Statistical significance is indicated as * *p* < 0.05, ** *p* < 0.01, and **** *p* < 0.001, when compared to untreated group.

To determine the MCE concentration that was safe to use in later experiments, B16F10 cells were incubated with the MCE at 0, 1, 5, 10, or 100 mg/mL, and cell viability was assessed. The viability of the cells incubated with the MCE at 5 mg/mL (91.00 ± 4.27%) or 10 mg/mL (97.00 ± 3.03%) did not significantly differ from that of the untreated cells (*p* < 0.05). However, when the MCE concentration was 100 mg/mL, the cell viability was reduced to 36.00 ± 3.00% (*p* < 0.001) (Figure 2A). Hence, 5 mg/mL and 10 mg/mL were the MCE concentrations selected for use in subsequent experiments.

Next, we assessed the effect of UVB radiation on B16F10 cells. Cell viability was unaffected at 30 and 60 mJ/cm^2^, while 90 mJ/cm^2^ significantly reduced cell viability to 62.00 ± 0.51% (*p* < 0.001) (Figure 2B). Therefore, 60 mJ/cm^2^ was used in the subsequent analyses. UVB doses of 30 and 60 mJ/cm^2^ resulted in significant increases in tyrosinase activity in B16F10 cells compared to no UVB radiation (150.00 ± 10.94% and 177.00 ± 12.49%, respectively; *p* < 0.05) (Figure 2C). Significant increases in the level of melanin were also observed after UVB exposure (160.00 ± 28.95% and 177.00 ± 20.94% at 30 and 60 mJ/cm^2^, respectively, *p* < 0.05), compared to the untreated group (Figure 2D).

### 2.4. Effect of the MCE on UVB Radiation-Induced Melanogenesis in B16F10 Cells

**Figure 3:**
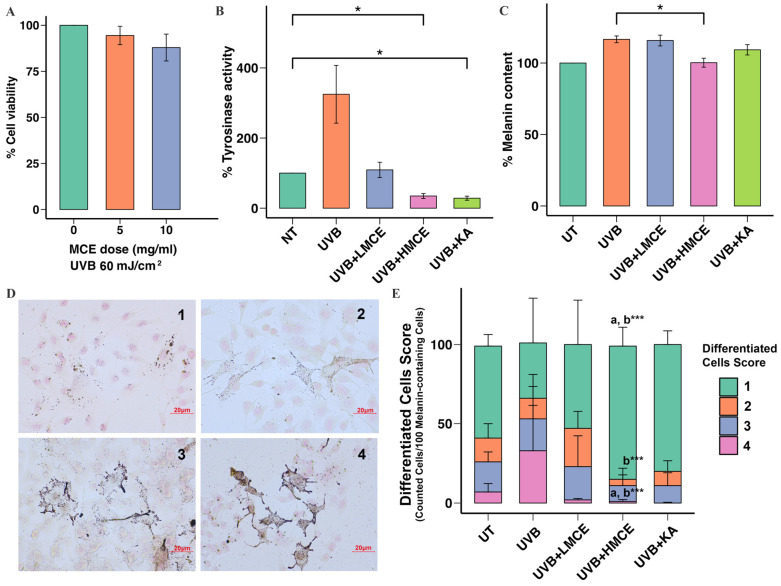
Inhibitory effect of MCE on UVB radiation-induced melanogenesis in B16F10 cells. B16F10 cells were exposed to UVB radiation (60 mJ/cm^2^) and treated with low-dose MCE (LMCE, 5 mg/mL) or high-dose MCE (HMCE, 10 mg/mL) for 24 h. (**A**) Cell viability, (**B**) tyrosinase activity, and (**C**) melanin content were assessed. (**D**) Representative images of pigmented cells scored from 1 (minimal pigmentation) to 4 (highly pigmented) (400× magnification). (**E**) Distribution of cells with different morphological features within and across treatment groups. Data are presented as mean ± standard deviation. * *p* < 0.05 and *** *p* < 0.01 indicate statistical significance between treatment groups. a and b indicate significant differences (*p* < 0.05) compared to UT and UVB radiation-only groups, respectively. UT represents untreated group.

To examine the capacity of the MCE to inhibit UVB radiation-induced melanogenesis, B16F10 cells were exposed to UVB radiation (60 mJ/cm^2^) and either the MCE at 5 mg/mL (low-dose MCE [LMCE]) or the MCE at 10 mg/mL (high-dose MCE [HMCE]) for 24 h. The viability of the cells exposed to the LMCE or the HMCE did not significantly differ from the viability of those in the control group (LMCE: 99.76 ± 6.15%, HMCE: 93.67 ± 5.76%) (Figure 3A).

The MCE was found to significantly inhibit tyrosinase activity in a dose-dependent manner. When compared to cells exposed to UVB radiation only, which showed 325 ± 67.29% activity, cells that were additionally incubated with the LMCE showed 109.00 ± 10.28% activity, while cells that were additionally incubated with the HMCE showed 35.00 ± 3.27% activity (Figure 3B). Regarding the impact of the MCE on the melanin content in cells exposed to UVB radiation, the LMCE did not significantly affect the melanin content; however, the HMCE significantly reduced the melanin content to 100.00 ± 1.84% (*p* < 0.01) (Figure 3C).

We analyzed the effects of the MCE on UVB radiation-induced changes and the distribution of melanin in B16F10 cells using Fontana-Masson staining and a scoring system. A total of 100 melanin-containing cells were evaluated and assigned a score of 1, 2, 3, or 4. Representative cells are shown in Figure 3D. As illustrated in Figure 3E, most of the cells in the control group were assigned scores of 1 and 2, while exposure to UVB radiation led to an increase in the number of cells assigned scores of 3 and 4 and a decrease in those scored 1 and 2 (*p* < 0.001). Treatment with the LMCE led to an increase in the number of cells assigned a score of 1 and 2 and a decrease in the number assigned a score of 3 and 4 compared to those exposed to UVB radiation only. Similar results were observed in the cells treated with the HMCE (*p* < 0.001). Cells exposed to UVB radiation and then incubated with kojic acid, which was used as a positive control, demonstrated the lowest proportion of cells scored as 4.

### 2.5. Effect of the MCE on UVB Radiation-Induced Melanogenesis in Zebrafish Embryos

**Figure 4:**
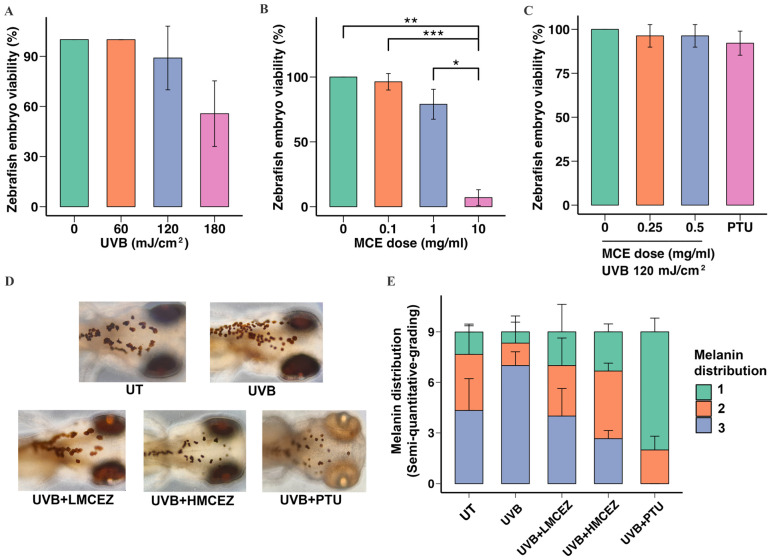
Inhibitory effect of MCE on UVB radiation-induced melanogenesis in zebrafish embryos. (**A**) Effect of UVB radiation only, (**B**) effect of MCE only, (**C**) and effect of UVB radiation combined with the MCE on zebrafish embryo viability. (**D**) Eye color, characteristics, and migration of melanocytes in head region after various treatments, observed via stereomicroscopy at 200× magnification. (**E**) Semi-quantitative scoring of melanin distribution, categorized as 1, 2, or 3. Data are presented as mean ± standard deviation. * *p* < 0.05, ** *p* < 0.01, and *** *p* < 0.001 indicate statistical significance between treatment groups. UT represents untreated group.

To determine a safe and effective radiation dose for inducing melanogenesis, we exposed embryos at 48 h post-fertilization to UVB radiation at doses of 60, 120, and 180 mJ/cm^2^. The results showed that the 60 and 120 mJ/cm^2^ doses did not induce mortality. However, embryos exposed to 180 mJ/cm^2^ exhibited a significant decrease in viability, with a survival rate of 56.00 ± 9.24% (*p* < 0.01) (Figure 4A). Zebrafish embryos were also treated with the MCE at 0.1, 1.0, and 10.0 mg/mL to determine a safe concentration. At 1.0 mg/mL, the MCE did not significantly affect mortality; viability was reduced to 79.00 ± 5.51% of that in the control group. In contrast, at 10.0 mg/mL, the MCE significantly reduced the viability to 7.00 ± 2.88% of that in the control group (Figure 4B).

We then exposed zebrafish embryos to 120 mJ/cm^2^ of UVB radiation and the MCE at either 0.25 mg/mL (low-dose MCE in zebrafish [LMCEZ]) or 0.50 mg/mL (high-dose MCE in zebrafish [HMCEZ]) or a positive control (0.1 mM phenylthiourea [PTU]) for 24 h before assessing the viability of the embryos using a stereomicroscope. As shown in Figure 4C, treating irradiated embryos with the LMCEZ or the HMCEZ did not significantly affect viability. In both cases, a viability rate of 96.30 ± 3.02% was recorded, which did not statistically differ from the rate recorded in the control group (*p* < 0.05).

Finally, we studied the effect of the MCE on UVB radiation-induced melanin distribution in zebrafish embryos, focusing on the head region. As shown in Figure 4D, irradiated embryos displayed a notable increase in the melanocyte migration compared to nonirradiated embryos, with melanocytes observed spreading from the head and exhibiting enhanced clustering and dendritic branches. Both the LMCEZ and HMCEZ inhibited melanocyte migration. In the embryos treated with the LMCEZ, melanocytes were morphologically similar to those observed in the control embryos, appearing as single, round cells. In contrast, treating embryos with the HMCEZ resulted in tiny, sparsely distributed melanocytes that resembled those in the embryos treated with PTU, which indicated limited connections and restricted migration. The results of the semi-quantitative analysis of the melanin distribution within and across the treatment groups are shown in Figure 4E. Most of the embryos that were exposed to UVB radiation only exhibited dark brown or black eye pigmentation and clusters of melanocytes and were thus assigned a score of 3. In contrast, among the irradiated embryos treated with the LMCEZ and HMCEZ, there were lower proportions of embryos that scored 3. In the group of irradiated embryos treated with PTU, no embryos were assigned a score of 3; most were scored 1, demonstrating characteristics such as gray-white eye color and single, disconnected melanocytes, with some being scored 2. There were no significant differences among the five treatment groups (ANOVA: F = 0.0013, *p* > 0.999). However, HMCEZ treatment was associated with a lower proportion of embryos scored 3, which suggested reduced melanin production in irradiated embryos treated with HMCEZ. Conversely, treating irradiated embryos with the LMCEZ resulted in morphological features and a melanin distribution pattern similar to those observed in the control embryos.

## 3. Discussion

In this study, we demonstrated that the tested MCE can suppress UVB radiation-induced melanogenesis in B16F10 melanoma cells and zebrafish embryos. Our results indicate that this anti-melanogenic activity is mediated by the inhibition of tyrosinase. In the zebrafish embryos, the MCE reduced the number and distribution of melanocytes, lightened their color, and altered their shape. Hence, this MCE could be a natural alternative to synthetic agents used to prevent or reduce hyperpigmentation caused by UV radiation.

Our finding that the tested MCE significantly reduced UVB radiation-induced tyrosinase activity and melanin content in B16F10 cells aligns with those of previous studies that demonstrated the inhibitory effects of mulberry extracts on tyrosinase activity and melanin formation [21,22,23]. It is well known that mulberries have a high antioxidant content, particularly anthocyanins, which possess potent free radical scavenging activity and play a crucial role in protecting the skin from the oxidative damage caused by UV radiation [16,29]. It is also thought that they inhibit tyrosinase activity [30]. In this study, the chromatographic analysis of the MCE showed that it contained several bioactive phytochemicals: rutin, chlorogenic acid, and mulberroside A. These compounds are known to possess strong antioxidant and anti-tyrosinase activities [31]. Rutin has been shown to inhibit melanin production by modulating the cellular oxidative status and inhibiting tyrosinase by directly chelating copper ions essential to tyrosinase function [32]. This chelation prevents tyrosinase from catalyzing the oxidation of tyrosine, the rate-limiting step of melanogenesis. Chlorogenic acid is an effective antioxidant known for its ability to reduce oxidative stress by scavenging ROS, thereby limiting tyrosinase activation [33]. It also exhibits anti-inflammatory properties, which further suppress UVB radiation-induced melanogenesis [34]. Mulberroside A is unique to mulberries and has demonstrated efficacy in reducing melanogenesis. It inhibits tyrosinase and reduces melanin transfer in the skin, thereby acting as a natural depigmenting agent [22]. Mulberroside A’s antioxidant and anti-inflammatory effects further support its capacity to combat UV radiation-induced pigmentation [22]. While tyrosinase inhibition accounted for a significant portion of the reduction in melanin production observed in this study, the MCE may have also exerted upstream regulatory effects through MITF. The MITF, a key regulator of melanocyte function, directly controls tyrosinase expression and is known to be upregulated by UVB radiation via the cyclic adenosine monophosphate, protein kinase A, and cyclic adenosine monophosphate response element-binding protein signaling cascade [35,36]. It has been reported that chlorogenic acid inhibits the expression of MITF, tyrosinase, TRP-1, and TRP-2 [37]. It is possible that the chlorogenic acid in the MCE may have attenuated this signaling pathway, which would have resulted in reduced MITF activation and the downregulation of tyrosinase. The potential synergistic effects among the multiple bioactive constituents in MCE may contribute to its overall anti-melanogenic activity. However, the specific mechanisms underlying these effects, as well as the individual and combined roles of the active compounds, remain to be elucidated. Further investigations, including compound isolation, characterization, and pathway-specific analyses, are warranted to better understand the mechanistic basis of MCE’s activity. It is also important to note that the initial screening using crude extracts in this study provides preliminary evidence of bioactivity, supporting the rationale for more detailed investigations. Crude extract-based screening serves not only as a cost-effective starting point but also as a valuable tool for gaining early insights into the extract’s therapeutic potential.

Regarding the overall safety of the MCE, no toxic or adverse developmental effects were observed in the zebrafish embryos treated with the MCE, even following prolonged exposure. Given the genetic and physiological similarities between zebrafish and humans, this model is widely used for preliminary toxicity screening of compounds designed for use in humans. The observed biocompatibility of the MCE reinforces its potential as a safe candidate for reducing UVB radiation-induced hyperpigmentation in clinical and cosmetic contexts. However, further testing in additional in vitro and in vivo models is necessary to confirm its safety and efficacy.

This study demonstrates the anti-melanogenic effects of mulberry crude extract (MCE) using both B16F10 cells and zebrafish embryos, enhancing its biological relevance. The combination of antioxidant assays, tyrosinase activity measurements, and phytochemical profiling supports its proposed mechanism of action. The use of zebrafish also provides preliminary safety data. However, the study did not explore molecular pathways such as MITF signaling, and quantification of active compounds was not performed. Additionally, the use of a single UVB dose, short exposure period, and lack of human skin models limit the generalizability of the findings. Further studies are needed to confirm long-term effects and clarify the underlying mechanisms involved.

In conclusion, our findings suggest that an extract derived from ripe white mulberry fruit may help inhibit tyrosinase activity and melanogenesis, indicating its potential as a natural candidate for mitigating UVB-induced hyperpigmentation. The observed antioxidant and anti-melanogenic properties provide preliminary support for its future exploration in cosmetic and dermatological applications.

## 4. Materials and Methods

### 4.1. Preparation of MCE

**Figure 5:**
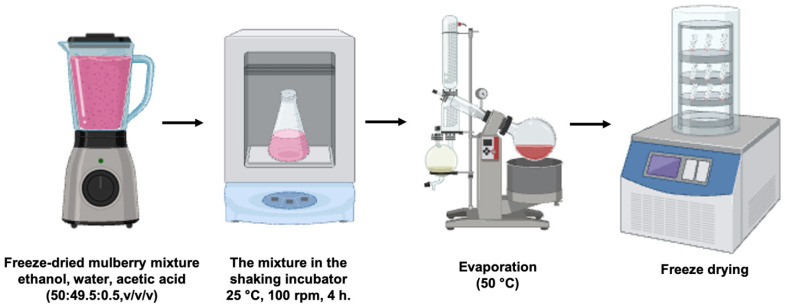
Schematic diagram of preparing mulberry crude extract (MCE).

Fresh, ripe white mulberry fruits were purchased from a reliable retailer in accordance with the United States Food and Drug Administration regulations. The extraction protocol was modified from that described by Li et al. [38]. As shown in Figure 5, we utilized a solvent mixture of ethanol, water, and acetic acid (50:49.5:0.5, *v*/*v*/*v*) due to its superior antioxidant and phenolic yields. Briefly, 500 g of freeze-dried fruit was blended with the solvent at a 1:2 (*w*/*v*) ratio for 30 s using a blender (Model HW-BDC2PC, 1500W, House Worth, Bangkok, Thailand). The mixture was incubated in aluminum foil-covered flasks at 25 °C for 4 h with shaking (100 rpm) (MaxQ™ 5000, Thermo Fisher Scientific, Waltham, MA, USA), centrifuged (2500 rpm, 25 °C) for 10 min (Heal Force Neofuge 18R, Heal Force Bio-Meditech Holdings Limited, Shanghai, China), and then filtered through Whatman No. 1 filter paper (Sigma Aldrich, Burlington, MA, USA). Ethanol was removed from the filtrate via rotary evaporation at 50 °C under reduced pressure (Buchi Rotavapor R200, Flawil, Switzerland), and the remaining water was removed by overnight freeze-drying (Supermodulyo-230, Thermo Fisher Scientific). The dried extract was stored at −80 °C. It was dissolved in distilled water type I and passed through a 0.45 µm PTFE membrane filter to maintain aseptic conditions prior to experimental use.

### 4.2. Assessment of Antioxidant Activity

#### 4.2.1. DPPH Radical Scavenging Assay

An equal volume of each sample (10–1000 μg/mL) was mixed with 100 μL of 160 μM DPPH solution and incubated in the dark at room temperature for 15 min. Absorbance was then recorded at 517 nm using an EMax Plus Microplate Reader (Molecular Devices, San Jose, CA, USA). DPPH radical scavenging activity was calculated as:

Scavenging (%) = [(A_0_ − Ac)/A_0_] × 100,

where A_0_ is the absorbance of the control and Ac is the absorbance of the sample. Antioxidant capacity was expressed as vitamin C equivalents per gram of extract.

#### 4.2.2. ABTS Radical Scavenging Assay

ABTS radical solution was prepared by mixing 7 mM ABTS with 2.45 mM potassium persulfate (1:1, *v*/*v*) and incubating in the dark at room temperature for 24 h. The solution was diluted with methanol to an absorbance of 0.70 ± 0.02 at 734 nm. Then, 50 μL of diluted ABTS radical was mixed with 50 μL of each sample (10–1000 μg/mL), incubated for 5 min in the dark, and the absorbance was measured at 734 nm. Scavenging activity was then calculated as:

ABTS scavenging activity (%) = ((A_0_ − Ac)/A_0_) × 100

where A_0_ and A_c_ are the absorbance of the control and sample, respectively. The results are expressed as the Trolox equivalent antioxidant capacity per gram of extract

#### 4.2.3. FRAP Assay

The FRAP reagent was prepared by combining 300 mM acetate buffer (pH 3.6), 10 mM TPTZ, and 20 mM FeCl_3_∙6H_2_O in a 10:1:1 ratio. Then, 175 μL FRAP reagent was added to 25 μL of each test sample at a concentration of 1000 μg/mL and incubated at 37 °C for 4 min. Ferrous sulfate (FeSO_4_∙7H_2_O) was used as the standard, and the absorbance was measured at a wavelength of 593 nm using a microplate reader. The results are expressed as millimoles (mmol) of FeSO_4_∙7H_2_O equivalents per gram of sample (mmol Fe^2+^/g).

### 4.3. Determination of Total Phenolic Content

The total phenolic content was assessed by the Folin–Ciocalteu assay. A 100 μL aliquot of each sample was combined with 200 μL of Folin–Ciocalteu reagent and left to react for 1 min. Then, 3 mL of 5% sodium carbonate solution was added, and the mixture was incubated in the dark at room temperature for 60 min. Absorbance was measured at 725 nm using a microplate reader. Gallic acid served as the reference standard, and phenolic content was calculated from a gallic acid calibration curve. Results are expressed as milligrams of gallic acid equivalents per gram of sample.

### 4.4. Predominant Phytochemical Fingerprint Analysis

The fingerprint of the phytophenolic profile of MCE was detected by a reverse-phase high-performance liquid chromatography (HPLC) (Waters Alliance e2695, Waters Corporation, Milford, MA, USA) with a UV-VIS detector (2489 UV-VIS Detector, Water, USA) and C18 column chromatography (SunFire C18 Column, 250 mm × 4.6 mm, Waters Corporation, USA). The protocols were adapted from the previous reports [23]. In brief, at room temperature, 10 μL of the sample was injected into a C18 column. Dissolvent A (0.4% aqueous acetic acid) and dissolvent B (acetonitrile) were contained in 1L bottle. The gradient condition of mobile phases (*v*/*v* of dissolvent A:B) is shown in Table 3. The mobile phase flow rate was set at 1.0 mL/min, and aliquots of 10 µL were injected for analysis. The detection wavelength was 324 nm.

The highest peak of absorbance was selected for UV-VIS detection in HPLC. The chromatogram of phytophenolic profiles was obtained at a flow rate of 1 mL/min for 60 min. The commercial standards of rutin, chlorogenic acid, and mulberroside A were applied as references. Compounds were detected at 324 nm, and the chromatogram was recorded over 60 min. Rutin (Tokyo Chemical Industry Co., Ltd., TYO, Japan; lot JJ57A-IB), chlorogenic acid (Sigma-Aldrich Chemie GmbH, Darmstadt, Germany; lot SLBS0103V), and mulberroside A (Chengu Biopurify Phytochemical Ltd., Chengdu, China) were used as reference compounds.

### 4.5. UVB Irradiation

A narrowband lamp tube (Philips TL 20W/01 RS; spectrum: 280–320 nm with a peak at 314 nm; Philips Lighting, Hansestadt Hamburg, Germany) was fixed to the chamber used as the radiator in this study. A light radiometer (model 850009, Sper Scientific, Scottsdale, AZ, USA) was used to calibrate the lamp before each experiment. The radiation intensity was 0.30 mW/cm^2^, and the time for exposure was determined based on a specific UV dose, calculated using the formula:

Time (seconds) = UV dose (mJ/cm^2^)/UV intensity (mW/cm^2^).

Irradiation was performed in a sealed radiation box using an open-lid cell culture plate coated with a thin layer of phosphate-buffered saline (PBS) [39]. The controls were shielded from UVB radiation.

### 4.6. Cell Culture and Treatment

The murine melanoma cell line B16F10 was obtained from the American Type Culture Collection (ATCC, Manassas, VA, USA, No. CRL-6475TM). The cells were cultured in low-glucose Dulbecco’s Modified Eagle Medium (DMEM) supplemented with 10% fetal bovine serum, 100 U/mL penicillin, and 100 mg/mL streptomycin at 37 °C in 5% CO_2_. The experimental treatment of the cells consisted of the following procedures. B16F10 cells were cultured at 1 × 10^5^ cells/well in 6-well plates and incubated at 37 °C in 5% CO_2_ for 24 h. The cells were then irradiated at a dose of 60 mJ/cm^2^ for 3.20 min and incubated at 37 °C in 5% CO_2_ for 3 h to allow for melanogenesis. After incubation, cells were treated with the MCE at 5 or 10 mg/mL for 24 h.

### 4.7. Cell Viability

Cell viability was assessed using a 3-(4,5-dimethylthiazol-2-yl)-2,5 diphenyltetrazolium bromide (MTT) assay. B16F10 cells were seeded in 96-well plates at 3 × 10^4^ cells/well and incubated for 24 h. The cells were then irradiated (60 mJ/cm^2^) and exposed to various concentrations of MCE before being incubated at 37 °C in 5% CO_2_ for a further 24 h. The cells were washed with PBS, and then MTT stock solution (0.5 mg/mL) (Sigma Aldrich) was added. The cells were then incubated for 3 h. The purple formazan crystals were dissolved in 100 µL of dimethyl sulfoxide (DMSO), and absorbance was measured at 405 nm using a microplate reader.

### 4.8. Cellular Tyrosinase Activity

Treated cells were washed three times with ice-cold PBS, lysed in 100 µL of PBS containing 1% Triton X-100 and 0.1 mM phenylmethylsulfonyl fluoride, and then frozen at −20 °C for 2 h. After thawing in a 37 °C water bath for 10 min, the cells were scraped and then centrifuged at 4 °C and 14,000 rpm for 15 min. The supernatant was transferred to a 96-well plate, and 20 mM 3,4-dihydroxy-L-phenylalanine (a tyrosinase substrate) was added. An orange-brown color indicated the production of dopachrome. Absorbance was measured at 492 nm every 15 min for 3 h using a microplate reader. Kojic acid, a natural compound known for its ability to inhibit tyrosinase, was used as a positive control.

### 4.9. Cellular Melanin Content

Melanin content in B16F10 cells was measured by absorption spectroscopy. Cells were lysed with 200 µL EDTA and 800 µL PBS, then centrifuged at 14,000 rpm and 4 °C for 15 min. The supernatant was removed, and the pellet was resuspended in PBS and centrifuged again. Next, 400 µL of 1 N NaOH containing 10% DMSO was added, and the mixture was heated at 80 °C for 1 h, with mixing every 15 min. After a final centrifugation, 200 µL of the clear yellow supernatant was placed in a 96-well plate for absorbance measurement.

### 4.10. Visualization of Cellular Melanin

Intracellular melanin was visualized using a Fontana-Masson Stain Kit (Bio-Optica, Milano, Italy). This staining method relies on melanin’s ability to reduce ammoniacal silver nitrate to metallic silver, resulting in black deposits. A nuclear fast red counterstain was applied to create a pinkish-red background. After fixation in 100% cold methanol for 5 min, treated samples were rehydrated with dechlorinated water, and staining was carried out following the manufacturer’s protocol.

### 4.11. Morphological Analysis of Cells Containing Melanin

Light microscopy (magnification: 100×) was used to assess specific morphological characteristics of treated cells found to contain melanin. For this, samples of 100 cells were evaluated. Each cell was scored as 1, 2, 3, or 4, according to the criteria shown in Table 4, and the proportions of each cell type in the sample were calculated.

### 4.12. Source and Maintenance of Parental Zebrafish

All protocols that involved the use of animals were approved by the Faculty of Science, Mahidol University Animal Care and Use Committee (MUSC65-029-622). Thirty parental zebrafish (15 males and 15 females) were sourced from the technology transfer center for ornamental fishes and aquatic plants, Department of Fisheries (DOF) in Pathum Thani, Thailand. They underwent a 1-week quarantine to minimize infection risk before being maintained in refiltered water at 27 ± 3 °C and pH 7.0 ± 1. The fish were subjected to a 14 h/10 h light/dark cycle and were fed twice daily with flake food supplemented weekly with live brine shrimp (Artemia salina). Stage of maintenance and all zebrafish-related experiments were handled in Pr109, the Zebrafish-Based Research in Aging Biology and Microbiome (ZAM) laboratory, Department of Pathobiology, Faculty of Science, Mahidol University.

### 4.13. Zebrafish Breeding and Embryo Harvesting

The 30 parental zebrafish were placed in a two-chambered 20 L acrylic mating tank (28.5 °C, pH 7.0 ± 1.0, 14 h/10 h light/dark cycle) to promote breeding. The lower chamber was screened off to prevent the adults from eating the eggs that fell into the lower chamber. After the 10 h dark period, the parental zebrafish were returned to the housing tank, and the embryos produced from the natural spawning events were collected within 30 min.

To evaluate the toxic effects of UVB radiation and MCE exposure, 10 embryos at 48 h post-fertilization were placed in a 96-well plate with 10% Hank’s Balanced Salt Solution. The embryos were irradiated (60–180 mJ/cm^2^) and exposed to various concentrations of the MCE (0, 0.1, 1.0, or 10.0 mg/mL). PTU, a chemical compound that inhibits tyrosinase, served as a positive control in the study using zebrafish to reduce pigmentation and enhance optical transparency for microscopic imaging. After incubation for 24 h at an ambient temperature, a stereomicroscope was used to evaluate mortality and morphological anomalies.

### 4.14. Semi-Quantitative Evaluation of Melanin Distribution in Zebrafish Embryos

The melanin distribution in the treated zebrafish embryos was evaluated using a semi-quantitative methodology. The embryos were classified into 3 groups based on eye color, which indicates melanin content, characteristics, morphology, and migration of melanocytes. The criteria are shown in Table 5.

### 4.15. Statistical Analysis

All data are presented as mean ± standard deviation values calculated from at least triple replicates. Student’s *t*-test was used to determine whether there were significant differences between groups. A one-way analysis of variance (ANOVA) was used for multiple comparisons among more than two groups. Results were considered statistically significant when *p* was <0.05.

## Figures and Tables

**Table 1:** Free radical scavenging activity and total phenolic content of MCE before and after it was passed through filter paper.

Parameter	Assay	Unfiltered MCE	Filtered MCE	*p*
Free radical scavenging activity	ABTS (mg Trolox/g sample)	32.00 ± 0.49	32.49 ± 0.16	0.13
	DPPH (mg vitamin C/g sample)	26.21 ± 0.55	25.45 ± 2.23	0.53
	FRAP (mmol Fe_2_SO_4_·7H_2_O/g sample)	45.66 ± 1.52	43.42 ± 2.33	0.37
Phenolic content	Folin–Ciocalteu (mg gallic acid/g sample)	2.20 ± 0.08	2.26 ± 0.13	0.46

Note. ABTS = 2,2′-azinobis-3-ethylbenzothiazoline-6-sulfonic acid (ABTS) assay, DPPH = 2,2-diphenyl-1-picrylhydrazyl (DPPH) assay, and FRAP = ferric reducing antioxidant power assay.

**Table 2:** Retention time (min), area under the curve (AUC), and quantity of some potential anti-melanogenic ingredients in MCE.

Active Ingredient	Reference Compound			MCE		
	Retention Time (min)	AUC	μg/mL	Retention Time (min)	AUC	μg/mL
Rutin	28.350	602,844	0.78	27.888	323,240	0.42
Mulberroside A	13.711	272,441	400.00	13.825	55,027	80.80
Chlorogenic acid	15.147	669,506	244.00	15.321	1,349,360	491.77

**Table 3:** Condition of gradient elution used for identifying active compounds in MCE.

Time (min)	Dissolvent A (%)	Dissolvent B (%)
0–20	95	5
20–30	85	15
30–35	80	20
35–40	45	55

**Table 4:** Criteria for scoring treated cells.

Criteria	Score
Bipolar dendrites, spindle-shaped cells 1–50 μm in size, <50% of cytoplasm pigmented, and low distribution of melanin.	1
Bipolar dendrites, spindle-shaped cells 1–50 μm in size, ≥50% of cytoplasm pigmented, and high distribution of melanin.	2
Multipolar dendrites without branches, size: >51 μm, and high distribution of melanin in the cytoplasm.	3
Multipolar dendrites with branches, size: >51 μm, and high distribution and condensed packing of melanin in the cytoplasm.	4

**Table 5:** Criteria for evaluating eye color and melanocytes in treated zebrafish embryos.

Criteria	Score
Gray-white eye color and single melanocytes (disconnected), round in shape.	1
Pale brown or red eye color and groups of melanocytes (2–3 connected cells), globular/bipolar in shape.	2
Brown or black eye color and clusters of melanocytes (3–5 connected cells) with expanded dendritic branches.	3

## Data Availability

All data produced or examined throughout this study have been incorporated into this published article.
